# Where You Live Matters: Neighborhood Environment and Dementia Risks Among U.S. Older Adults

**DOI:** 10.1093/geroni/igaf122.891

**Published:** 2025-12-31

**Authors:** Haowei Wang

**Affiliations:** Syracuse University, Syracuse, New York, United States

## Abstract

Alzheimer’s Disease and Related Dementias (ADRD) are a growing public health crisis among older adults. Emerging research suggests that neighborhood environment plays a significant role in ADRD risks. This study explores the association between various aspects of neighborhood environment and the risk of ADRD in the US older adult population. We used a large scale, nationally representative data from the All of Us research program, including N = 15,683 respondents aged 60+. Respondents provided information about their neighborhood environment and their ADRD diagnosis was accessed using Electric Health Records. Using factor analysis, we identified four aspects of neighborhood environment, including physical environment disorders, accessibility of facilities, relationship with neighbors, and neighborhood safety. We estimated structural equation models (SEM) with latent variables to analyze the relationship between ADRD risk and multiple aspects neighborhood environment, controlling for respondents’ sociodemographic and behavioral characteristics. SEM results showed that older adults had higher risks of ADRD diagnosis if there were more environmental disorders and fewer accessible facilities in the neighborhood. Perceived neighborhood safety was also associated with a lower risk of ADRD diagnosis among older adults. Understanding how neighborhood environments contribute to ADRD risk is crucial for developing targeted public health interventions and promoting healthy cognitive aging for older adults in the US.

